# Extrarenal Clinical Features are Reported for Most Genes Implicated in Genetic Kidney Disease

**DOI:** 10.1016/j.ekir.2025.01.045

**Published:** 2025-02-05

**Authors:** Benjamin Serrano, Judy Savige

**Affiliations:** 1 The University of Melbourne Department of Medicine, Melbourne Health and Northern Health, Royal Melbourne Hospital, Victoria, Australia

**Keywords:** CAKUT, ciliopathies, extrarenal features, genetic kidney disease, proteinuric kidney disease, tubulopathies

## Abstract

**Introduction:**

Genetic kidney disease is often suspected based on a family history of the disease or the presence of extrarenal features. This study examined how often a positive family history or syndromic features are found.

**Methods:**

A total of 255 genes from the Genomics England “green” lists for congenital anomalies of the kidney and urinary tract (CAKUT) (*n* = 57), ciliopathies and cystic kidney diseases (*n* = 90), hematuria (*n* = 5), renal proteinuria (*n* = 55), and renal tubulopathies (*n* = 48) were examined for mode of inheritance and, in the Online Mendelian Inheritance in Man (OMIM), for reported clinical features in different systems (neurological, cardiac, etc.) that would be obvious on a history or physical examination.

**Results:**

Autosomal recessive (AR) inheritance was recorded for 148 of the 248 genes (60%) with an OMIM entry. Extrarenal features were associated with 221 genes (89%), including those causing hematuria (5, 100%), renal ciliopathies (86, 97%), CAKUT (52, 91%), renal tubulopathies (41, 85%), and proteinuric renal diseases (37, 76%).The median number of affected systems was 4 (range: 0–10). More extrarenal features were associated with CAKUT (4, 0–10) and the ciliopathies (5, 0–9) than with hematuria (2, 2–5), proteinuria (3, 0–7), and the tubulopathies (3, 0–7) (*P* < 0.00001). The most commonly-affected systems were growth and musculoskeletal (164, 66%), neurological (147, 59%), and ocular (133, 54%).

**Conclusion:**

Extrarenal associations have been reported for most genes affected in genetic kidney disease, and are more common with pediatric-onset conditions with recessive inheritance. However, information is limited for how often extrarenal features are found in any individual.

## Graphical abstract

**Figure:**
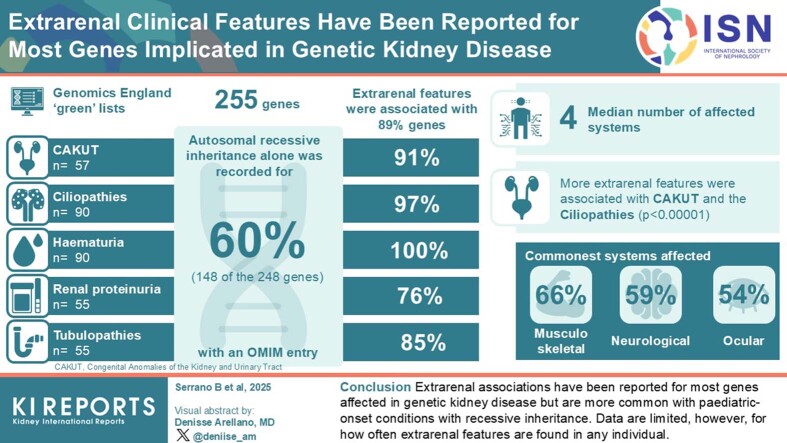

Genetic kidney disease is common, accounting for at least 50% of children and 20% of adults with end-stage kidney failure.^1^ It is important to recognize the genetic nature of kidney disease because early diagnosis has implications for treatment, prognosis, reproductive decision-making, and family screening.^2^^,^^3^ However, many genetic diseases remain undiagnosed.^4^^,^^5^

Genetic kidney disease is suspected when the phenotype is typically genetic, such as for the ciliopathies, cystic kidney disease, and for many forms of CAKUT. Genetic diseases are also suspected in patients with a positive family history. However, there are many instances where there is no family history, for example, where inheritance is AR or biallelic, disease is *de novo*, the family is very small, the history is not known, or where inheritance is X-linked (XL) and the family comprises only women.^6^ In addition, clinical features and diagnoses may vary in different family members with the same disease.^7^ Often, genetic kidney disease is suspected when disease onset is in childhood^3^ but, increasingly, “pediatric-onset” diseases are also recognized for the first time in adults.

Genetic kidney disease is also suspected when extrarenal or syndromic manifestations are present. These may be obvious on physical examination but sometimes the association with genetic disease is not recognized. These features are not only useful diagnostically, but may have implications for patients, such as the hearing loss in Alport syndrome, intracranial aneurysms in autosomal dominant (AD) polycystic kidney disease, and diabetes and infertility in AD tubulointerstitial kidney disease–*HNF1B*. Some features require ongoing monitoring or urgent management, such as repair of a cardiac anomaly.^8^ Others impair the patient’s quality of life for decades because of successful treatment with dialysis or transplantation.

Extrarenal features may not be identified until “reverse phenotyping” or closer examination of the patient occurs after the genetic test results are known and the association is recognized. A review of the expected extrarenal features after genetic diagnosis may also help differentiate between multiple variants of uncertain significance in a person with the characteristic clinical phenotype.

Extrarenal manifestations often affect the eyes. Ocular abnormalities may be obvious on physical examination, so they are particularly helpful.^9^, ^10^, ^11^, ^12^ A coloboma-like disc may be present in *PAX2-*related disease in CAKUT and focal segmental glomerulosclerosis, and lenticonus and fleck retinopathy are pathognomonic for XL and AR Alport syndrome.^13^ Other extrarenal features affect the musculoskeletal system, head and neck, hearing, and heart. Demonstration of extrarenal features in chronic kidney disease increases the diagnostic yield of exome sequencing in adult patients.^3^ However, both kidney disease and extrarenal manifestations may vary greatly, for the same gene and even for the same variant.^3^

Despite the recommendation to consider genetic kidney disease when there is a positive family history or extrarenal features, little information is available on how many genetic kidney disease–associated genes have extrarenal manifestations and which organ systems are affected. This study reviewed the genes recommended for testing in suspected genetic kidney disease in the Genomics England panels for the mode of inheritance (and thus the likelihood of an affected family member) and for reported extrarenal features that suggested a genetic basis.

## Methods

### Modes of Inheritance and Extrarenal Manifestations in Major Phenotypes of Genetic Kidney Disease

Genes were downloaded from the Genomics England gene panels (https://panelapp.genomicsengland.co.uk) for CAKUT; for both the renal ciliopathies and cystic kidney disease (which overlap); and for hematuria; proteinuric renal disease; and the renal tubulopathies. Genes were selected from the “green” lists of the panels, which were associated with a renal phenotype by an expert panel, based on conclusive evidence of an association in 3 families, or in 2 families with additional experimental evidence. The lists were accessed on December 5, 2023.

The modes of inheritance for each gene-associated disease were recorded directly from the Genomics England database. The OMIM website (www.omim.org) was consulted for the extrarenal clinical features for the genes. These were extracted directly from the corresponding clinical synopses and recorded under the headings of growth and musculoskeletal; skin, hair, nails; head and neck; neurological; ocular; hearing; cardiovascular; respiratory; gastrointestinal and liver; and genitalia. Extrarenal disease was assessed only on the clinical features of these systems that were apparent on history, physical examination, or routine testing. The numbers of systems affected were expressed as the median and range for genes where there was an OMIM entry. Differences between the reported number of affected systems were examined using the Mann-Whitney U test. The overall likelihood of organ involvement was expressed as the percentage of all genes for the kidney phenotype where there was an OMIM entry.

### Modes of Inheritance and Extrarenal Manifestations in Most Common Causes of Genetic Kidney Disease

Data for the genes affected in the most common causes of genetic kidney disease were reviewed for modes of inheritance and extrarenal manifestations likely to be found most often.^14^, ^15^, ^16^

## Results

### Genetic Kidney Diseases

A total of 255 genes that were affected in the following genetic kidney diseases were examined: CAKUT (*n* = 57), renal ciliopathies (*n* = 75), cystic kidney disease (*n* = 32, 17 in both renal ciliopathies and cystic kidney disease, with a combined total of 90), hematuria (*n* = 5), proteinuric renal disease (*n* = 55), and renal tubulopathies (*n* = 48) (Table 1; Supplementary Table S1). Seven genes (3%) had no entries in OMIM (*DLG5* in renal ciliopathies and cystic kidney diseases; and 6 in the proteinuric kidney diseases [*APOE, DLC1, FAT1, ITSN1, PODXL* and *TNS2*]) and were excluded. The following results refer to the 248 genes with OMIM entries.

**Table 1:** Modes of inheritance and extrarenal manifestations of genetic kidney disease

	Phenotypes with clinical features in OMIM					
Feature	CAKUT (*n* = 57)	Renal ciliopathies (*n* = 75) plus cystic kidney disease (*n* = 32); 17 in both	Hematuria (*n* = 5)	Proteinuric renal disease (*n* = 55)	Renal tubulopathies (*n* = 48)	All
Total number of genes in GE panel (s)	57	90	5	55	48	255
Number of genes with no entry in OMIM	None	One *(DLG5)*	None	Six *(APOE, DLC1, FAT1, ITSN1, PODXL, TNS2)*	None	7
Number of genes examined in OMIM	57	89	5	49	48	248
Number of genes with extrarenal features	52 (91%)	86 (97%)	5 (100%)	37 (76%)	41 (85%)	221 (89%)
Genes with no reported extrarenal features in OMIM	5 *(BNC2, DSTYK, MYOCD, ROBO2, TBX18)*	3 *(DZIP1L, MAPKBP1, UMOD)*	None	12 *(ACTN4, COQ8B, DAAM2, DGKE, FN1, MAGI2, MYO1E, NPHS2, NUP93, PLCE1, TBC1D8B, TRPC6)*	7 *(CUL3, KLHL3, MAGED2, SLC22A12, SLC2A9, UMOD, WNK4)*	27 (11%)
AD only	25 (44%)	13 (15%)	2 (40%)	10 (20%)	12 (25%)	62 (25%)
AR only	23 (40%)	70 (79%)	0	31 (63%)	24 (50%)	148 (60%)
AD and AR	4 (7%)	4 (4%)	2 (40%)	2 (4%)	10 (21%)	22 (9%)
X-linked	5 (9%)	2 (2%)	1 (20%)	6 (12%)	2 (4%)	16 (6%)
Median number of reported extrarenal systems	4 (0 -10)	5 (0 -10)	2 (2 -5)	3 (0 -7)	3 (0 -7)	4 (0 – 10)
Number of genes with extrarenal features affecting this system						
Growth and musculoskeletal	35 (61%)	68 (76%)	1 (20%)	25 (51%)	35 (73%)	164 (66%)
Skin, nails and hair	20 (35%)	17 (19%)	2 (40%)	9 (18%)	8 (17%)	56 (23%)
Head and neck	42 (74%)	47 (53%)	1 (20%)	17 (35%)	10 (21%)	117 (47%)
Neurological	27 (47%)	67 (75%)	1 (20%)	26 (53%)	26 (54%)	147 (59%)
Hearing	12 (21%)	9 (10%)	4 (80%)	12 (24%)	6 (13%)	43 (17%)
Ocular	28 (49%)	66 (74%)	5 (100%)	26 (53%)	8 (17%)	133 (54%)
Cardiovascular anomalies	23 (40%)	37 (42%)	1 (20%)	7 (14%)	15 (31%)	83 (33%)
Respiratory	21 (37%)	37 (42%)	0	2 (4%)	5 (10%)	65 (26%)
Gastrointestinal and liver	30 (53%)	53 (60%)	0	12 (24%)	22 (46%)	117 (47%)
Genitalia	26 (46%)	34 (38%)	1 (20%)	3 (6%)	2 (4%)	66 (27%)
Hematological	1 (2%)	6 (7%)	2 (40%)	10 (20%)	9 (19%)	28 (11%)
Endocrine and metabolic	11 (19%)	18 (20%)	0	4 (8%)	36 (75%)	69 (28%)
Immunological	2 (4%)	2 (2%)	0	3 (6%)	3 (6%)	10 (4%)

AD, autonomic dominant; AR, autonomic recessive; GE, Genomics England; OMIM, Online Mendelian Inheritance in Man; CAKUT, congenital anomalies of the kidney and urinary tract. The chromosomal regions were not included separately. Renal ciliopathies and cystic kidney disease were combined because 17 genes were found in both.

### Mode of Inheritance

For the genes considered here, inheritance was AR (148, 60%), AD (62, 25%), both AD and AR (22, 9%), or XL (16, 6%) (Table 1). AD variants were most common in CAKUT, and AR variants in the other genetic kidney diseases. XL disease represented 1 in 5 (20%) of the genes affected in the hematuria panel but otherwise < 10% of the genes affected in genetic kidney disease.

### Extrarenal Features

Twenty-seven genes (11%) had no reported extrarenal features in the OMIM database. These were in the categories for CAKUT (*BNC2, DSTYK, MYOCD*), ciliopathies and cystic kidney disease (*DZIP1L, MAPKBP1*), renal proteinuria (*ACTN4,APOE, COQ8B, DAAM2, FN1, MAGI2, MYO1E, NPHS1, NUP107, NUP93, PLCE1, PODXL, TBC1D8B, TRPC6)* and the tubulopathies (*MAGED2, SLC22A12, SLC2A9)*.

**Figure 1:**
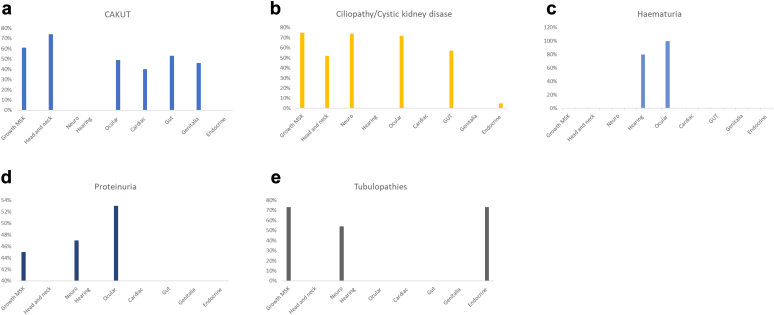
Extrarenal systems affected in Genomics England Gene panels for (a) congenital anomalies of the kidney and urinary tract,(b) ciliopathies/cystic kidney diseases, (c) hematuria, (d) proteinuria, and (e) the tubulopathies. The % on the vertical axis refers to how often individual systems were affected in this type of genetic kidney disease. Endocrine and metabolic effects in the ciliopathy and cystic kidney disease panel refer to hyperuricemia in autonomic dominant tubulointerstitial kidney disease caused by *UMOD* pathogenic variants.

Most genes affected in genetic kidney disease (221/248, 89%) had several reported extrarenal manifestations (Table 1, Figure 1, Supplementary Tables S1–S5). Extrarenal features were common with all kidney phenotypes and were found with genes affected in hematuria (5, 100%), the ciliopathies and cystic diseases (86, 97%), CAKUT (52, 91%), the tubulopathies (37, 76%), and proteinuric kidney diseases (41, 85%).

The median number of extrarenal systems affected was 4 (range: 0–10). More extrarenal features were associated with CAKUT (4, 0–10), the ciliopathies and cystic kidney disease (5, 0–10) than for hematuria (2, 2–6), proteinuric renal disease (2, 0–6) and the renal tubulopathies (3, 0–7) (*P* < 0.00001).

Overall, the most common extrarenal systems affected were growth and musculoskeletal (164, 66%), neurological (147, 59%), ocular (133, 54%), gastrointestinal and liver (117, 47%), and head and neck (117, 47%).

#### CAKUT

The most common mode of inheritance for genes affected in CAKUT was AD (25, 44%) (Table 1). Most affected genes (52, 91%) had extrarenal manifestations. The most commonly affected systems were head and neck (42, 74%), growth and musculoskeletal (35, 61%), gastrointestinal and liver (30, 53%), genitalia (26, 46%), and cardiac (23, 40%) systems. The most common manifestations in each of these systems were microcephaly (15, 26%), short stature (10, 18%), constipation (7, 12%), cryptorchidism (15, 26%), and ventricular septal defect (14, 24%) (Table 2).

**Table 2:** Most common features from each disease category

Disease category	Most common organs or systems involved	Most common features from each system
CAKUT (*n* = 57)	Head and Neck (*n* = 42, 74%) Growth and musculoskeletal (*n* = 35, 61%) Gastrointestinal (*n* = 30, 53%) Ocular (*n* = 28, 49%) Genitalia (*n* = 26, 46%) Cardiovascular (*n =* 23, 40%)	Microcephaly (*n =* 15, 26%) Short stature (*n =* 10, 18%) Cryptorchidism (*n =* 15, 26%) Constipation (*n =* 7, 12%) Ventricular septal defect (*n =* 14, 24%)
Ciliopathies and cystic kidney diseases (*n =* 89)	Neurological (*n =* 66, 74%) Growth and musculoskeletal (*n =* 67, 75%) Ocular (*n =* 64, 72%) Gastrointestinal (*n =* 51, 57%) Head and neck (*n =* 46, 52%)	Polydactyly (*n =* 45, 51%) Molar tooth sign (*n =* 23, 26%) Inherited retinal degeneration (*n =* 21, 24%) Low-set ears (*n =* 18, 20%)
Hematuria (*n =* 5)	Ocular (*n =* 5, 100%) Hearing (*n =* 4, 80%)	Cataracts (*n =* 5, 100%) Hearing loss (*n =* 4, 80%)
Proteinuric renal disease (*n =* 49)	Ocular (*n =* 26, 53%) Neurological (*n =* 23, 47%) Growth and musculoskeletal (*n =* 22, 45%)	Seizures (*n =* 15, 31%) Short stature (*n =* 9, 18%) Cataracts (*n =* 7, 14%)
Tubulopathies (*n =* 48)	Endocrine (*n =* 35, 73%) Growth and musculoskeletal (*n =* 35, 73%) Neurological (*n =* 26, 54%)	Failure to thrive (*n =* 17, 35%) Seizures (*n =* 17, 35%) Metabolic acidosis (*n =* 17, 35%)

CAKUT, congenital anomalies of the kidney and urinary tract.

#### Renal Ciliopathies and Cystic Kidney Diseases

AR was the most common mode of inheritance (70,79%). Overall, 86 of the 89 affected genes (97%) were associated with extrarenal manifestations. The commonest affected systems were growth and musculoskeletal (68, 76%), neurological (67, 75%), ocular (66, 74%), gastrointestinal and liver (53, 60%), and head and neck (47, 53%) (Table 1). The most common manifestations of these systems were polydactyly (45, 51%), molar tooth sign (23, 26%), inherited retinal degeneration (21, 24%), hepatic fibrosis, and low-set ears (18, 20%), respectively **(**Table 2**)**.

#### Hematuria

The inheritance modes were AD only (2), AD with AR (2), and XL (1). All 5 genes in the hematuria panel were associated with extrarenal manifestations. The commonest affected extra-renal systems were ocular (5 of 5, 100%) and hearing (4 of 5, 80%) (Table 1). The most common manifestations were cataracts (5, 100%) and hearing loss (4, 80%) (Table 2).

#### Proteinuric Kidney Disease

The most common mode of inheritance was AR (31, 63%). Overall, 37 of the 50 genes studied (76%) were associated with extrarenal manifestations (Table 1). The commonest affected extrarenal systems were ocular (26, 53%), neurological (26, 53%), and growth and musculoskeletal (25, 51%). The most common manifestations were cataracts (5, 100%), seizures (15, 31%), and short stature (9, 18%)(Table 2).

#### Tubulopathies

The most common mode of inheritance was AR (24, 50%). Forty-one of the 48 genes (85%) were associated with extrarenal manifestations (Table 1). The commonest affected extrarenal systems were growth and musculoskeletal (35, 73%), and neurological (26, 54%). The most common manifestations were failure to thrive (17, 35%) and seizures (17, 35%). Endocrine and metabolic effects were also common (36% and 75%, respectively), including metabolic acidosis (17, 35%) (Table 2).

### Affected Systems

The clinical features associated with the genes in each disease type are found in the Supplementary Material, and a summary of the most important features is provided here.

#### Growth and Musculoskeletal

Clinical features varied from short stature and failure to thrive to skeletal abnormalities, such as polydactyly. Overall, the commonest abnormalities were polydactyly and short stature. Polydactyly was associated most often with genes affected in the ciliopathies and cystic kidney diseases (45, 51%). Short stature was associated most often with CAKUT (10, 18%) and renal proteinuria (9, 16%) (Table 2).

#### Neurological

The most common neurological abnormalities were intellectual disability or delay, and seizures. Intellectual disability or delay was associated with 61 genes (25%) overall, but was not more frequently associated with any kidney phenotype. Seizures were associated with the tubulopathies (17, 35%) and renal proteinuria (15, 31%).

#### Head and Neck

The most common head and neck abnormalities were low-set ears (36, 15%) and microcephaly (35, 14%). Low-set ears were most common with the ciliopathies and cystic kidney diseases (18, 20%). Microcephaly was mainly found associated with CAKUT (20, 35%).

#### Ocular

The most common ocular abnormalities were hypertelorism (28, 11%) and nystagmus (24, 10%); however, these were not found more frequently with any kidney phenotype. Inherited retinal degeneration was common in ciliopathies and cystic kidney diseases (21, 24%). Cataracts were observed in all disease types and were associated with all 5 hematuria genes.

#### Gastrointestinal and Liver

The most common gastrointestinal abnormalities were feeding difficulties (21, 8%), hepatomegaly (17, 7%), and hepatic fibrosis (17, 7%). Hepatic pathology was commonest in the genes affected in the ciliopathies and cystic kidney diseases with hepatic fibrosis (14, 16%) and hepatomegaly (11, 12%).

#### Genitalia

Genital abnormalities were most common in the CAKUT, and ciliopathies and cystic kidney disease panels. The most common genital abnormality was cryptorchidism (15, 26%).

### Conditions not Commonly Associated With Genetic Kidney Disease

The results of testing for hematological, endocrine (diabetes and thyroid disease), metabolic, and immunological abnormalities were uncommon associations of genetic kidney disease.

### Extrarenal Manifestations of the Most Common Genetic Kidney Diseases

Of the 21 genes that were most commonly affected in the 5 major kidney phenotypes,^14^, ^15^, ^16^ inheritance was AD in 12, AD and AR in 2, AR in 5, and XL in 2. All of these genes were represented in OMIM, and 16 (76%) had extrarenal features (Table 3). However, the 2 most commonly affected genes (*COL4A3* and *COL4A4*) have no extrarenal associations in AD Alport syndrome but are associated with hearing loss and ocular abnormalities in the much less common AR Alport syndrome.

**Table 3:** Clinical manifestations of the most common genetic kidney diseases

Kidney disease and affected genes	Kidney disease	Inheritance	Growth and musculoskeletal	Skin, nails and hair	Head and neck	Neurological	Hearing	Ocular	Cardiac	Respiratory	Gastrointestinal and liver	Genitalia
CAKUT												
*EYA1*	Branchiootorenal syndrome 1	AD	YES		YES		YES	YES				
*GATA3*	Hypoparathyroidism, sensorineural deafness and renal dysplasia	AD					YES					YES
*PAX2*	Papillorenal sydnrome	AD	YES	YES		YES	YES	YES				
*PBX1*	CAKUT without hearing loss	AD	YES	YES	YES	YES	YES	YES	YES	YES	YES	YES
*SALL1*	Townes-Brocks syndrome 1	AD	YES		YES	YES	YES	YES	YES		YES	YES
Ciliopathies and cystic kidney diseases												
*PKD1*	AD polycystic kidney disease	AD							YES		YES	
*PKD2*	AD polycystic kidney disease	AD			YES				YES			
ARPKD - *PKHD1*	AR polycystic kidney disease	AR			YES					YES	YES	
ADTKD -*HNF1B*	AD tubulointerstitial kidney disease	AD	YES			YES					YES	YES
ADTKD - *UMOD*	AD tubulointerstitial kidney disease	AD	YES									
Haematuria												
*COL4A5*	XL Alport syndrome	XL					YES	YES				
*COL4A3 or COL4A4*	AD Alport syndrome	AD or AR	No extrarenal features reported (Hearing loss and ocular abnormalities occur only in the rarer AR Alport syndrome)									
Renal proteinuria												
*NPHS1*	Nephrotic syndrome type 1	AR	YES									
*NPHS2*	Nephrotic syndrome type 2	AR	No extrarenal features reported									
*LAMB2*	Pierson syndrome	AR	YES			YES		YES				
*TRPC6*	FSGS type 2	AD	No extrarenal features reported									
*GLA*	Fabry disease	XL	YES			YES			YES	YES	YES	
*INF2*	Glomerulosclerosis 5; Charcot-Marie-Tooth syndrome	AD	YES			YES	YES					
*ACTN4*		AD	No extrarenal features reported									
Tubulopathies												
*SLC12A3*	Gitelman syndrome	AR	YES			YES			YES		YES	

AD, autonomic dominant; AR, autonomic recessive; CAKUT, congenital anomalies of the kidney and urinary tract; FSGS, focal segmental glomerulosclerosis.

## Discussion

These results indicate that extrarenal anomalies are associated with most genes affected in genetic kidney disease, especially those causing CAKUT, and the ciliopathies and cystic kidney diseases. A median of 4 extrarenal features have been reported that are potentially found with a thorough history and examination. Anomalies in growth and the musculoskeletal, neurological, and ocular systems are the most commonly affected systems. Previous studies have differentiated between genetic and “syndromic” renal disease but nearly all genes involved in genetic kidney disease are sometimes associated with extrarenal features and thus syndromic.^17^

This study considered the genes affected in 5 major genetic kidney phenotypes but the conclusions were also similar for the 21 genes affected most often in adults with genetic kidney disease. Although inheritance was more likely to be AD in adults, the majority (16/21, 75%) still had extrarenal associations, including the most common genetic causes of end-stage kidney failure (XL Alport syndrome, AD polycystic kidney disease and AD tubulointerstitial kidney disease -*UMOD* or -*HNF1B*).^14^, ^15^, ^16^^,^^18^, ^19^, ^20^

The most common genetic kidney diseases found in adults have AD inheritance (CAKUT, AD polycystic kidney disease, and AD Alport syndrome),^20^, ^21^, ^22^ where other affected family members are already recognized. The present study demonstrated that, overall, more genetic kidney diseases have AR inheritance and pediatric onset with extrarenal manifestations. However, these pediatric diseases are increasingly diagnosed in adults, where extrarenal features occur less often. In addition, phenotypes are now often recognized for the heterozygous or carrier forms of AR kidney diseases that demonstrate AD inheritance, but have milder renal disease with fewer, if any, extrarenal features. Genes for which both AR and AD forms occur include *COL4A3 and COL4A4; NPHP1;* and *NEK8*.^23^, ^24^, ^25^ This means that while extrarenal features are helpful in suggesting a genetic basis for kidney disease, they may be less useful in adults than in children.

One of the limitations of this study is that the data examined do not indicate how often anomalies are associated with each gene, and some may have been reported only once in a single individual. In addition, published clinical reports are sometimes incomplete because of inadequate examination or because the information was not available to the laboratory. Nevertheless, the demonstration of a median of 4 extrarenal features in the history and examination for all genetic kidney diseases suggests that finding at least 1 extrarenal feature is likely and potentially useful in identifying the genetic basis.

This study found that a median of 4 extrarenal features were associated with genes affected in genetic kidney disease. These were all obvious in the history or examination, such as short stature, skeletal abnormalities, facial anomalies, ocular defects, and hearing loss. The effects on growth may be at least partly because of the consequences of kidney failure, but other skeletal abnormalities, such as polydactyly in ciliopathies and cystic kidney disease, are also common. Recognizable facial features may be highly informative for clinicians and even analyzable from photographs.^26^ Further anomalies require hematological, endocrine, metabolic, or immunological testing for their demonstration. Overall, the skin, hair, and nails were affected least.

Almost any extrarenal abnormalities suggest genetic kidney disease. Age at presentation may be an important factor, with some features present from birth and others evident only in adulthood. Some are present even before kidney disease becomes obvious, whereas others are obvious only after the development of kidney failure.

Many clinical features will be obvious from the patient’s referral letter (atrial septal defect, diabetes) or the patient may demonstrate an anomaly themselves (finger webbing, dislocatable patella). Retinal imaging may be useful.^9^, ^10^, ^11^, ^12^ Some features are more important to identify, such as hearing loss. Some may be difficult to fit into the working diagnosis, but genetic testing is conclusive and distinguishes the disease from phenocopies. Some features may only be obvious on reverse genotyping.^27^

In addition, phenotypes may vary, even for a single genetic variant. Furthermore, renal phenotypes overlap, and although the presentation may be with proteinuria, the gene may be associated with CAKUT, kidney cysts, tubulopathy, or all 4 phenotypes, as occurs with *HNF1B-*nephropathy.

The presence of extrarenal features suggests a genetic basis for kidney disease that should be confirmed with genetic testing.^7^ Sometimes, the clinical features are specific enough to indicate the affected gene and, hence, the diagnosis.

The involvement of extrarenal tissues probably occurs in genetic kidney disease because most affected genes encode proteins that are also expressed in tissues other than the kidney.^7^ Extrarenal features were found most often associated with genes affected in CAKUT, the ciliopathies and cystic kidney diseases, as well as the 5 genes affected in hematuria. CAKUT often results from pathogenic variants in transcription factors. Proteins encoded by the CAKUT genes are expressed in ectodermal tissues where the skin and skeleton are affected, which is reflected in nearly two-thirds of the CAKUT genes being associated with musculoskeletal abnormalities.^28^ In contrast, ciliated cells are critical signal transduction mediators in the central nervous system and retina,^10^^,^^29^ and many ciliopathies are associated with neurological, developmental, and ocular abnormalities. The genes affected in Alport syndrome encode the principal components of the basement membranes in the glomerulus, cochlea, and eye,^30^ which explains the renal, hearing, and ocular associations.^30^^,^^31^ The genes affected in tubulopathies often encode renal tubular transporters^32^ which result in perturbed acid-base homeostasis, failure to thrive, and defective growth. In contrast, the genes involved in renal proteinuria affect the proteins expressed in podocytes, especially the cytoskeleton,^33^ which are possibly expressed less often or at lower levels in other tissues.^34^

The strengths of this study were the use of Genomics England panels to identify genes associated with different renal phenotypes and the extraction of standardized clinical data from OMIM. These gene panels include genes examined in genetic testing for major renal phenotypes in the UK and elsewhere.

The major limitation of this study was that it was not possible to determine how frequently extrarenal features occurred in association with any gene because this information was not included in OMIM and is rarely reported. In addition, some clinical features were only described in 1 affected family, and sometimes, in only a single family member. This study was not a literature review but rather examined the Genomics England gene lists in OMIM to determine the modes of inheritance of genetic kidney disease and how often the affected genes are associated with extrarenal features. Occasional genes, such as *NPHP1* and *TSC* were missing from the list, and sometimes well-recognised clinical features were absent. Thus, head and neck manifestations were listed for *PKD2* but not *PKD1*. Nevertheless, this study had sufficient data to confirm that extrarenal features are common in genetic kidney disease, and, in general, we did not add to or modify the data.

In conclusion, extrarenal features are common and may be used to confirm the genetic basis of kidney disease and sometimes suggest the diagnosis and affected gene. Individuals with suspected genetic kidney disease should undergo thorough history-taking and physical examinations to identify extrarenal manifestations. These are especially useful when a family history is lacking. Many extrarenal features require clinical evaluation, treatment or monitoring. However, the absence of extrarenal manifestations does not exclude genetic disease, but their presence should prompt genetic testing. Future studies should examine the likelihood of extrarenal manifestations in individual genetic kidney diseases.

## Disclosure

All the authors declared no competing interests.

## Supplementary Material

Supplementary File (PDF) **Table S1.** Systems affected in CAKUT. **Table S2.** Systems affected in the ciliopathies and cystic kidney disease**. Table S3.** Systems affected in hematuria-associated genes. **Table S4.** Systems affected in proteinuric renal disease. **Table S5.** Systems affected in the renal tubulopathies.

## Footnotes

Supplementary File (PDF)
**Table S1.** Systems affected in CAKUT. **Table S2.** Systems affected in the ciliopathies and cystic kidney disease. **Table S3.** Systems affected in hematuria-associated genes. **Table S4.** Systems affected in proteinuric renal disease. **Table S5.** Systems affected in the renal tubulopathies.
