# Erratum to “Extrarenal Clinical Features are Reported for Most Genes Implicated in Genetic Kidney Disease” [*Kidney International Reports* Volume 10, Issue 4, April 2025, Pages 1196-1204]

**DOI:** 10.1016/j.ekir.2025.05.029

**Published:** 2025-05-17

**Authors:** Benjamin Serrano, Judy Savige

**Affiliations:** 1 The University of Melbourne Department of Medicine, Melbourne Health and Northern Health, Royal Melbourne Hospital, Victoria, Australia

In the original published version of this article, the following errors were introduced during the copyediting process by the Suppliers.

The text originally published “More extrarenal features were associated with CAKUT (4, 0–10), the ciliopathies and cystic kidney disease (5, 0–10) than for hematuria (2, 2–6), proteinuric renal disease (2, 0–6) and the renal tubulopathies (3, 0–7) (*P* < 0.00001).”

Has been corrected to “More extrarenal features were associated with CAKUT (4, 0–10) plus the ciliopathies and cystic kidney disease (5, 0–10) than for haematuria (2, 2-6) plus proteinuric renal disease (2, 0-6) and the renal tubulopathies (3, 0–7) (*P* < 0.00001).”

The text originally published “The commonest affected extra-renal systems were ocular (5 of 5, 100%) and hearing (4 of 5, 80%) (Table 1). The most common manifestations were cataracts (5, 100%) and hearing loss (4, 80%) (Table 2).”

Has been corrected to “The commonest affected systems were ocular (5/5, 100%), and hearing (4/5, 80%) (Table 1). The commonest manifestations of these systems were cataracts (5, 100%) and hearing loss (4, 80%) respectively (Table 2).”

The text originally published “The most common genetic kidney diseases found in adults have AD inheritance (CAKUT, AD polycystic kidney disease, and AD Alport syndrome),^20–22^ where other affected family members are already recognized.”

Has been corrected to “The commonest genetic kidney diseases found in adults have AD inheritance (CAKUT, ADPKD and AD Alport syndrome)^20–22^ where another affected family member is already recognized.”

The text originally published “The most common manifestations of these systems were polydactyly (45, 51%), molar tooth sign (23, 26%), inherited retinal degeneration (21, 24%), hepatic fibrosis, and low-set ears (18, 20%), respectively”

Has been corrected to “The commonest affected systems were head and neck (42, 74%), growth and musculoskeletal (35, 61%), gastrointestinal and liver (30, 53%), genitalia (26, 46%), and cardiac (23, 40%)”

The text originally published “Endocrine and metabolic effects were also common (36% and 75%, respectively), including metabolic acidosis (17, 35%)”

Has been corrected to “Endocrine and metabolic effects were also common (36, 75%), including metabolic acidosis (17, 35%)”

These errors bear no reflection on the article or its authors. The publisher apologizes to the authors and the readers for this unfortunate error.

DOI of original article: https://doi.org/10.1016/j.ekir.2025.01.045

